# Size-Dependent Electrochemical and Morphological Properties of Magnetite Nanoparticles Adsorbed on Electrodes

**DOI:** 10.1021/acsmeasuresciau.5c00014

**Published:** 2025-04-07

**Authors:** Gayan Premaratne, Silan Bhandari, Charuksha Walgama, Bhaskara V. Chikkaveeraiah, Albert Jin, Sadagopan Krishnan

**Affiliations:** † Department of Chemistry, 7618 Oklahoma State University, Stillwater, Oklahoma 74078, United States; ‡ Department of Physical & Applied Sciences, University of Houston-Clear Lake, 2700 Bay Area Boulevard, Houston, Texas 77058, United States; § Laboratory of Cellular Imaging and Macromolecular Biophysics, National Institute of Biomedical Imaging and Bioengineering, National Institutes of Health, Bethesda, Maryland 20892, United States

**Keywords:** magnetite nanoparticles, particle size effects, electrochemical analysis, peroxidase-like activity, microscopy, measurement interface, nanostructured electrodes

## Abstract

We investigated the influence of particle size on the electrochemical behavior of Fe_3_O_4_ magnetite nanoparticles (MNPs) electrostatically adsorbed onto graphite electrodes modified with a preadsorbed poly­(ethylenimine) polycation layer. Three hydrodynamic sizes (50, 100, and 200 nm) were selected to assess size-dependent differences in electrochemical response using cyclic voltammetry under well-controlled adsorption and measurement conditions. The 50 nm MNPs exhibited the highest electroactive response and peroxidase-like electrocatalytic currents, which are consistent with greater surface area-to-volume ratios. Qualitative image analysis from atomic force microscopy and scanning electron microscopy revealed closer particle spacing and more extended surface contact for the smaller MNPs, in contrast to isolated aggregates formed by larger particles. These surface-level differences were reflected in the electrochemical signals, where the 50 nm particles yielded higher electroactive surface coverage. The study demonstrates how particle size and interfacial organization influence electrochemical readouts, underscoring the utility of correlating microscopy with electrochemical data to evaluate nanoparticle-based sensing interfaces.

## Graphical abstract

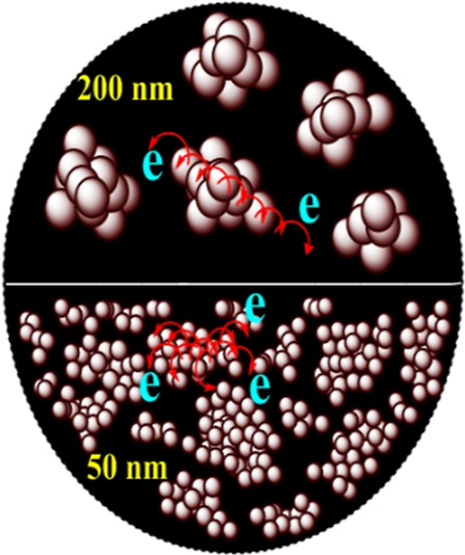

## 1 Introduction

Understanding the size-dependent electrochemical properties of redox-active nanomaterials is crucial for advancing applications in electronics and catalysis. Among these, Fe_3_O_4_ magnetite nanoparticles (MNPs) have garnered significant attention for their unique physicochemical, electronic, and catalytic properties.^1–5^ These nanoparticles are widely utilized in sensitive biosensors, in vitro and in vivo biomedical applications, analyte separation and preconcentration, and catalysis, making them a versatile material for various fields.^6,7^

Magnetite (Fe_3_O_4_) exhibits an inverse spinel structure, [Fe^3+^]_A_[Fe^2+^,Fe^3+^]_B_[O^2–^]_4_, and a half-metallic behavior at room temperature,^8^ where one spin channel exhibits metallic conductivity, and the other acts as a semiconductor or insulator. This high spin polarization at the Fermi level makes Fe_3_O_4_ a promising candidate for electrochemical applications.^9^ Additionally, magnetite nanoparticles have intrinsic peroxidase-like activity and electron mediation capabilities, which are advantageous for sensing and catalytic processes.^10–13^ Despite these benefits, the direct evaluation of size-dependent electron transfer properties of bare MNPs electrostatically adsorbed on electrodes remains largely unexplored.

Previous studies have investigated related aspects of Fe_3_O_4_ electrochemistry. Lecuire analyzed the redox behavior of iron oxides using crystalline powders in graphite paste.^14^ Leddy and Scherer et al. reported the redox potentials of magnetite suspensions under reducing conditions and characterized such materials electrochemically.^15,16^ Similarly, Encinas et al. characterized the structural and chemical properties of magnetite through the electrochemical behavior of Fe­(II) and Fe­(III) compound mixtures.^17^ More recently, Roberts et al. examined the electrochemistry of 4 nm magnetite nanoparticles, revealing slower heterogeneous electron transfer rates due to mass transfer limitations.^18^ Dong et al. demonstrated the intrinsic electron mediation capabilities of Fe_3_O_4_, facilitated by the Fe­(II)–O–Fe­(III) chain, but noted phase transformations to γ-Fe_2_O_3_ during extended catalysis, which reduced catalytic efficiency.^19^

In this study, we systematically investigated the size-dependent electrochemical properties of Fe_3_O_4_ MNPs with hydrodynamic sizes ranging from 50 to 200 nm. The nanoparticles were negatively charged due to poly­(acrylic acid) functionalization and electrostatically adsorbed onto polyethylenimine (PEI)-coated high-purity graphite (HPG) electrodes (Scheme 1, conceptual representation of this report). The optimal concentration and volume conditions were determined to enable efficient electron transport between the iron centers of MNPs and the electrode surface.

**Scheme 1:**
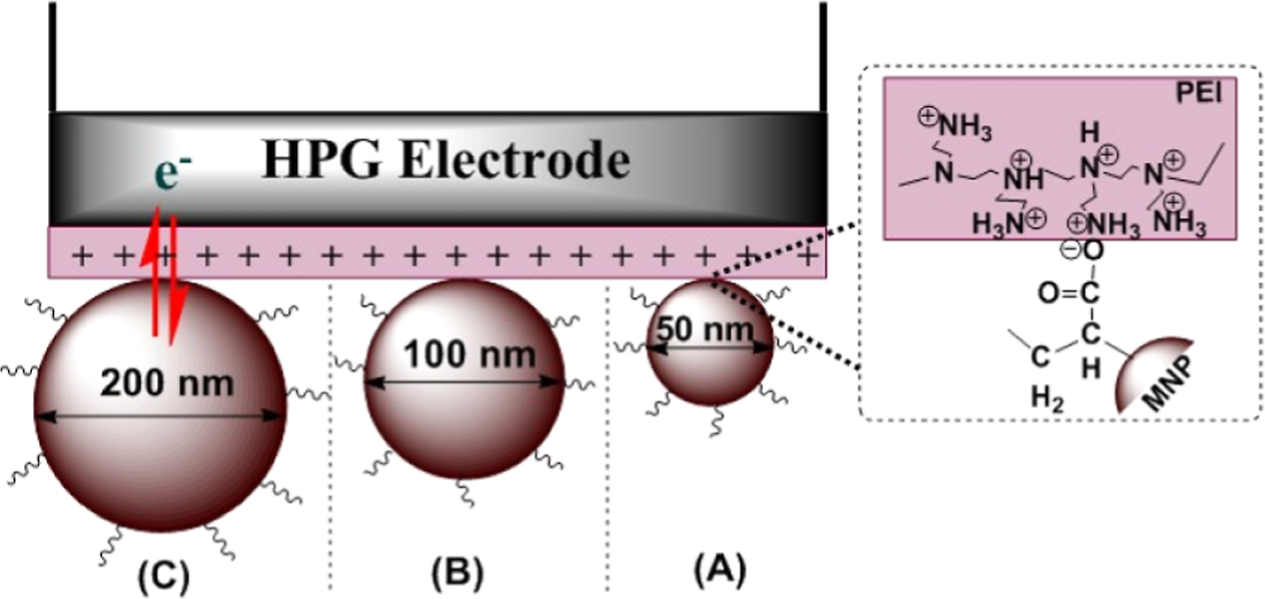
Conceptual Representation of Polyacrylic Acid-Functionalized Fe_3_O_4_ Magnetite (A) 50, (B) 100, or (C) 200 nm MNPs Electrostatically Immobilized on Polyethyleneimine (PEI) Adsorbed High-Purity Graphite Disk Electrodes.

## 2 Experimental Section

### 2.1 Materials and Methods

Magnetite nanoparticles with hydrodynamic sizes of 50, 100, and 200 nm were purchased from Chemicell Inc. (Berlin). In our study, these particles were coated with a hydrophilic polymer, specifically poly­(acrylic acid).^20–22^ These particles were characterized in our laboratory for the hydrodynamic size and zeta potential using a ZetaPALS potential analyzer (Brookhaven Instruments Corporation, Holtsville, NY, USA) (Table S1, Supporting Information). The particles, with hydrodynamic diameters of 100–200 nm, have a multidomain core and can be separated by an external magnetic field using a magnet. In contrast, MNPs with a particle size of 50 nm consist of a single domain core. Hence, they are not easily separable by an external magnetic field. According to the supplier, the number of MNPs per gram was 1.3 × 10^16^ particles for 50 nm, 1.8 × 10^15^ for 100 nm, and 2.2 × 10^14^ for 200 nm hydrodynamic sizes. To electrostatically adsorb the negatively charged magnetite nanoparticles (MNPs) onto a positively charged polymeric layer, we used poly­(ethylenimine) (PEI) modified high-purity graphite disk electrodes (HPG, geometric area 0.2 cm^2^). The PEI solution (product no. 408179, *M*_w_ ∼ 800 and *M*_n_ ∼ 600) was purchased from Sigma-Aldrich, and the HPG electrodes were sourced from McMaster-Carr (Atlanta, GA). A phosphate-buffered solution (pH 7.0) was used as the electrolyte. All other chemicals and reagents used in this study were of analytical grade and high purity.

In this study, HPG electrodes were used as the working electrodes. Prior to each experiment, the electrodes were polished on SiC-grit paper (P320), sonicated for 30 s, rinsed with deionized (DI) water, and dried with ultrapure nitrogen gas. To prepare the electrodes, 20.0 μL of a poly­(ethylenimine) (PEI) solution (1 mg mL^–1^) was placed on freshly polished HPG electrodes. The electrodes were then incubated for 15 min in a cold and humid environment to prevent the polymer from drying during the adsorption process. Following incubation, the electrodes were rinsed with DI water to remove any unbound PEI molecules. This methodology of preparing polyion-modified graphite electrodes was similar to our prior reports.^23–25^

Following the PEI adsorption, aliquots of magnetite nanoparticle suspensions (2 mg mL^–1^) with sizes of 50, 100, or 200 nm were electrostatically adsorbed onto the PEI-coated HPG electrodes. The electrodes were incubated for 20 min under cold, moist conditions to ensure the effective attachment of the nanoparticles. Various volumes of the 2 mg mL^–1^ MNP suspensions were studied to determine the optimum surface adsorbed condition that offered high-quality cyclic voltammograms. Afterward, the electrodes were rinsed with DI water and subjected to cyclic voltammetric studies in a phosphate-buffered solution (pH 7.0). Cyclic voltammetry was performed using a CHI 6017 potentiostat, with an Ag/AgCl electrode (1 M KCl) as the reference electrode and a platinum wire as the counter electrode. In all CV measurements, the forward scan proceeded from the initial potential toward the negative potential region, while the reverse scan moved toward the positive potential region.

Electrocatalytic studies were performed using freshly prepared MNP-adsorbed, PEI-modified HPG electrodes for each substrate. These studies were conducted in a saturated oxygen (O_2_) buffer or an argon-purged anaerobic buffer solution containing 0.5 mM hydrogen peroxide (H_2_O_2_) using rotating disc voltammetry (RDV) at an electrode rotation rate of 500 rpm. Morphological characterization of the MNPs was carried out using multimode pico-force atomic force microscopy (AFM, Bruker, CA),^26^ and scanning electron microscopy (SEM, FEI Quanta 600FE).^27,28^

## 3 Results and Discussion

### 3.1 Electrochemical Measurements

#### 3.1.1 Anaerobic Cyclic Voltammetry in Argon Atmosphere

##### 3.1.1.1 Concentration and Volume Optimization

To obtain well-defined cyclic voltammetric peaks for all MNPs, optimal volumes of a 2 mg mL^–1^ MNP suspension in water were identified for adsorption onto polycation film-coated electrodes (Figure 1). The optimal volumes were 10.0 μL for 50 nm, 5.0 μL for 100 nm, and 2.5 μL for 200 nm MNP suspensions, with an electrode geometric area of 0.2 cm^2^.

**Figure 1:**
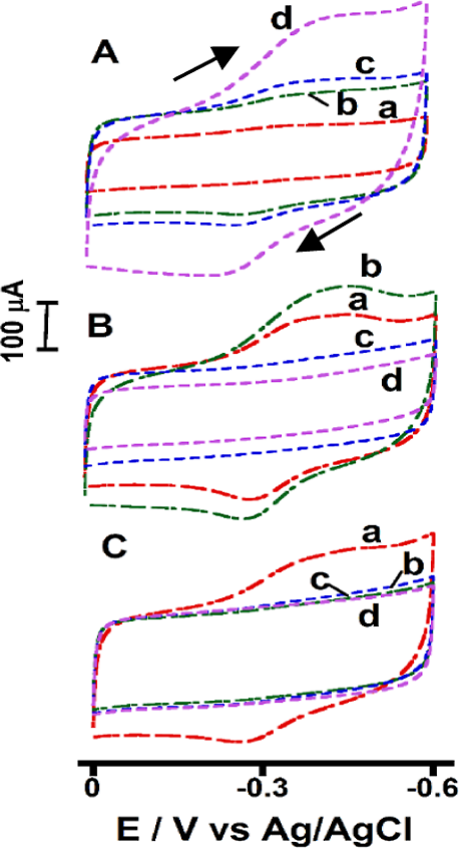
(A) 50, (B) 100, and (C) 200 nm MNP suspensions (2 mg mL^–1^) adsorbed onto a PEI layer on graphite electrodes. In each plot, (a) 2.5, (b) 5.0, (c) 7.5, and (d) 10.0 μL MNP suspensions were used, scan rate 1.0 V s^–1^, phosphate buffer solution (pH 7.0), saturated argon, 25 °C.

For the 50 nm MNPs, the current increased with the number of particles adsorbed onto the electrodes from the higher concentration suspensions. This observation indicates enhanced electronic connectivity between the MNP iron centers and the electrode, likely facilitated by an increase in the number of MNP-to-electrode surface contacts for charge transfer (Figure 1A). In contrast, for the 100 and 200 nm MNPs, increasing the number of particles adsorbed led to a decrease in peak currents (Figure 1B,C). The higher surface area-to-volume ratio of the smaller 50 nm MNPs likely enhanced the electroactivity. For the larger MNPs, their lower surface area of contact with the electrode surface and reduced interconnectivity likely caused lower currents with increasing particle adsorption. Microscopic studies were conducted to gain further insights into proposed possibilities, as discussed in the subsequent sections. Magnetite exhibits an inverse spinel structure, with Fe­(III) ions occupying the tetrahedral sites and an equal distribution of Fe­(II) and Fe­(III) ions at the octahedral sites. The rapid electron hopping between Fe­(II) and Fe­(III) ions at the octahedral sites results in an intermediate +2.5 valence state, which contributes to the conductivity and half-metallic behavior of magnetite at room temperature.^29^ The observed MNP voltammograms align with existing literature, including studies on the electrochemistry and dissolution kinetics of magnetite,^30^ as well as the solution voltammetry of 4 nm magnetite iron oxide nanoparticles.^18^

### 3.2 Particle Size-Dependent Electroactivity at the Optimal Condition

Cyclic voltammetry of MNP-adsorbed, PEI-modified graphite electrodes under anaerobic conditions (argon) exhibited quasi-reversible redox peaks corresponding to the iron centers of the MNPs (Figure 2a–c). In contrast, bare electrodes coated with only a PEI layer showed no peaks in the examined potential region (Figure 2d). The forward reduction peaks were broader than the reverse oxidation peaks, likely due to the reduction of two Fe­(III) centers with differing geometries during the forward scan. The sharper oxidation peaks in the reverse scan suggest a structural reorganization of the MNPs, facilitating a more thermodynamically favorable process.^31,32^

**Figure 2:**
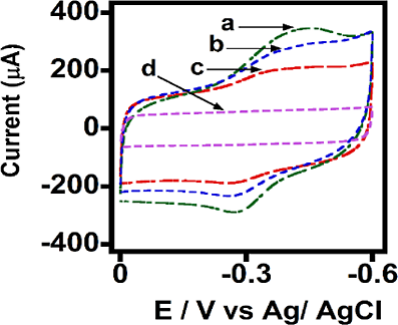
Comparative cyclic voltammograms of electrostatically adsorbed films of MNPs on polycation adsorbed HPG electrodes at the optimal volume of MNP suspensions (2 mg mL^–1^) derived in Figure 1. (a) 50 nm MNPs, (b) 100 nm MNPs, (c) 200 nm MNPs, and (d) polycation background voltammogram in phosphate-buffered solution (pH 7.0, 0.1 M KCl), Scan rate 1.0 V s^–1^, saturated argon, 25 °C.

Moreover, the forward reduction peak (*E*_p,red_) shifted more negatively with increasing scan rates (Figure 3), while the shift in the reverse oxidation peak (*E*_p,ox_) was minimal to moderate. For instance, as the scan rate increased from 0.1 to 1 V s^–1^, the *E*_p,red_ shifted by −38, −40, and −15 mV, for electrodes with 50, 100, and 200 nm MNPs, respectively. In comparison, the corresponding *E*_p,ox_ shift ranged from 8 to 20 mV. These observations suggest that the reduction process at the Fe centers of the MNPs is kinetically limiting, while the reverse oxidation process is kinetically faster, resulting in smaller shifts in peak potential with increasing scan rates.

**Figure 3:**
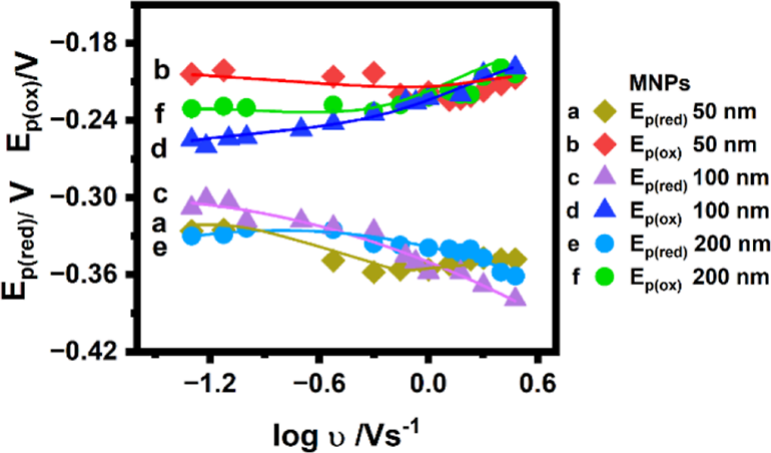
Forward scan reduction and reverse scan oxidation peak potentials as a function of the logarithm of the scan rate for electrostatically adsorbed MNPs on PEI-modified HPG electrodes. (a,b) 50 nm MNPs, (c,d) 100 nm MNPs, and (e,f) 200 nm MNPs in phosphate-buffered solution (pH 7.0, 0.1 M KCl), saturated with argon, 25 °C.

For all MNP films, the peak currents increased linearly with scan rates (Figure 4A–C), indicating that the direct electron transfer process is surface-confined.

**Figure 4:**
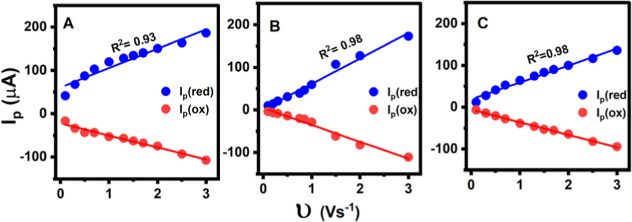
Reduction and oxidation peak currents (*I*_p_) vs scan rate (υ): (A) 50, (B) 100, and (C) 200 nm MNPs adsorbed on PEI-modified HPG electrodes in phosphate-buffered solution (pH 7.0), saturated with argon, 25 °C.

### 3.3 Electroactive Amount

The integration of the area under the cyclic voltammetry forward peak revealed that smaller MNPs exhibited higher electroactive amounts compared to larger hydrodynamic MNPs. At a scan rate of 1 V s^–1^, the electroactive amounts at the optimal conditions were 326 ± 11 pmol cm^–2^ for 50 nm, 119 ± 4 pmol cm^–2^ for 100 nm, and 55 ± 3 pmol cm^–2^ for 200 nm MNPs. This suggests that smaller particles provide greater access to Fe centers due to their higher surface-to-volume ratio.

### 3.4 Estimation of the Percentage of Electroactive Fe Centers

The percentage of electroactive Fe centers was determined by comparing the ratio of electrochemically detected Fe to the total number of Fe atoms used in the adsorbing MNP suspensions on the electrode. The amount of Fe atoms on the electrode was calculated based on the manufacturer’s information on the number of particles per gram and the face-centered cubic (fcc) lattice structure of magnetite (Table 1). The percentage of active Fe centers was determined by comparing the initial MNP amount in the prepared suspension placed on electrodes for adsorption and the electrochemically detected electroactive Fe from the forward peak area integration. This parameter is critical for assessing the efficiency of electrochemical activity. The analysis revealed that smaller particles had a higher fraction of active Fe centers, which was attributed to their improved accessibility and enhanced electrochemical activity. This finding infers the importance of optimizing particle size and suspension concentration to maximize electrode performance (Table 1).

**Table 1:** Electrochemically Estimated Fe on Polyacrylic Acid-Covered Magnetic Nanoparticles (MNPs) Adsorbed onto Polyethyleneimine-Modified Electrodes.

size (nm)	concentration (mg/mL)	volume (μL)	mass (g)	particles (per g)	Fe atoms per geometric electrode area (pmol/cm^2^)	electrochemically detected Fe (pmol/cm^2^)	% active Fe centers
50	2	10	2.00 × 10^–5^	1.3 × 10^16^	9.6 × 10^5^	326	0.034
100	2	5	1.00 × 10^–5^	1.8 × 10^15^	5.3 × 10^5^	119	0.022
200	2	2.5	5.00 × 10^–6^	2.2 × 10^14^	2.6 × 10^5^	55	0.021

### 3.5 Microscopic Insights into Observed Electroactivity

Figure 5A–C presents the atomic force microscopy (AFM) images of MNPs dry-coated on a mica surface. The smaller MNPs (50 nm) exhibited more interconnected aggregation across the surface compared to the larger particles, which formed more isolated clusters with larger unoccupied electrode areas. As the particle size increased from 50 to 200 nm, the surface coverage became less uniform, with larger particles forming distinct clusters and leaving more vacant surface areas. Mica was chosen as the substrate for AFM imaging due to its atomically flat and smooth surface, which extends over macroscopic areas.^33^ Unlike the rough and surface-defective solid graphite disks, mica provides a superior platform for identifying particle arrangements and height differences in AFM with improved resolution. This allowed for a clearer understanding of MNP spatial distribution and its relation to the observed electroactivity on electrodes, thus providing an indirect analysis.

**Figure 5:**
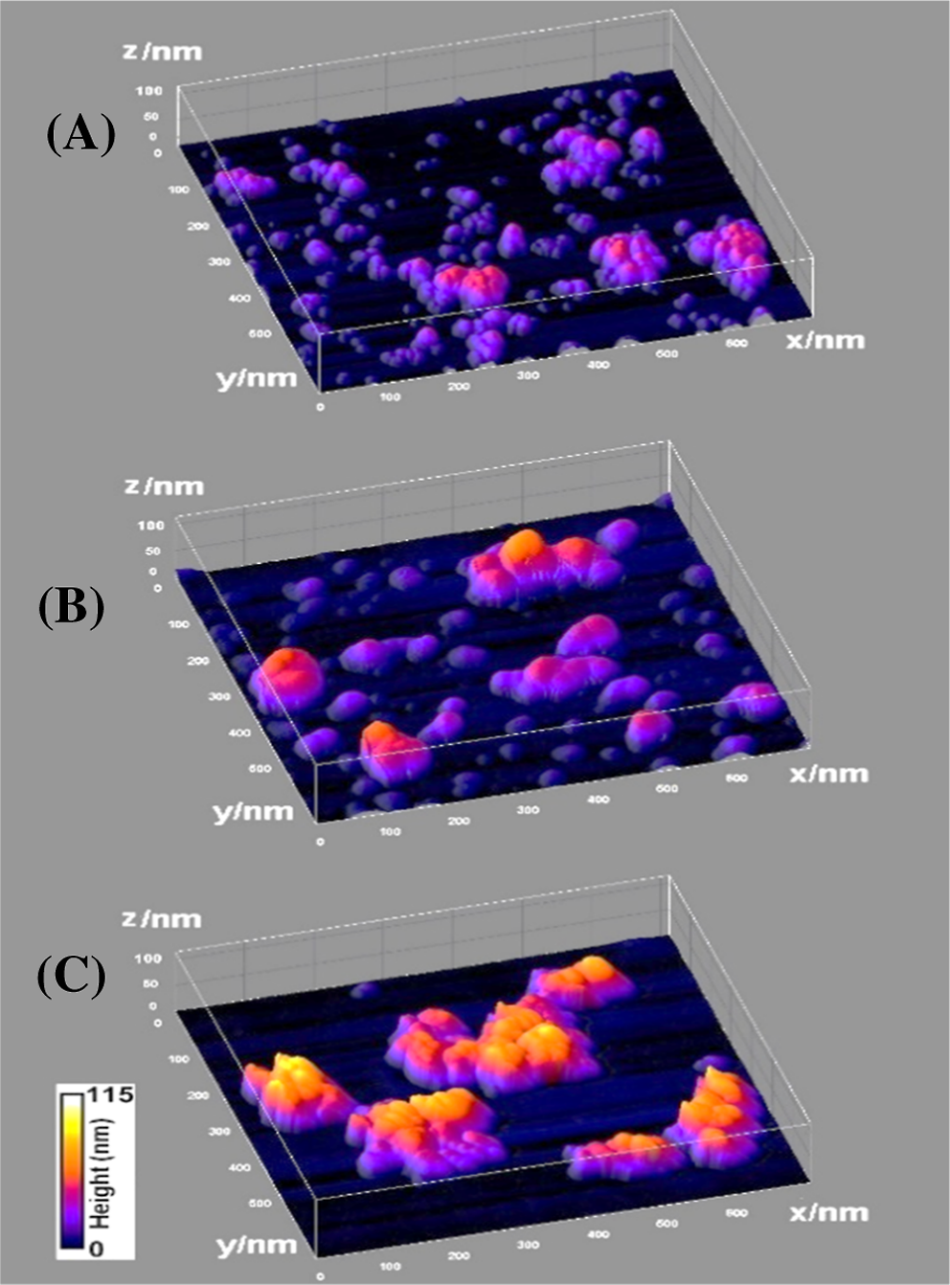
AFM surface morphologies of dry-coated MNPs on a mica surface: (A) 50, (B) 100, and (C) 200 nm hydrodynamic diameters. The AFM-measured heights are significantly lower than the respective hydrodynamic particle diameters in solution, due to the imaging analysis of dried sample nanoparticles with a cluster appearance.

Scanning electron microscopy (SEM) images of MNPs electrostatically adsorbed onto PEI-coated HPG electrodes (Figure 6A–D) revealed differences in particle aggregation and surface coverage based on particle size. The 100 and 200 nm MNPs formed more isolated clusters, leaving larger areas of the electrode surface unoccupied. In contrast, the 50 nm MNPs showed greater interparticle connectivity, with fewer free electrode spaces between aggregates. These observations suggest that the smaller 50 nm particles could facilitate enhanced electron hopping^29^ due to their higher connectivity and greater electrode-to-particle contact. In comparison, the larger particles, due to their isolated clustering, reduce both interparticle interactions and the contact of the electrode surface for electron transfer. Thus, the SEM results, conducted directly on the designed MNPs adsorbed PEI-adsorbed HPG electrodes, correlate with the AFM analysis on the mica surface, adding rigor to the interpretation of the electrochemical findings.

**Figure 6:**
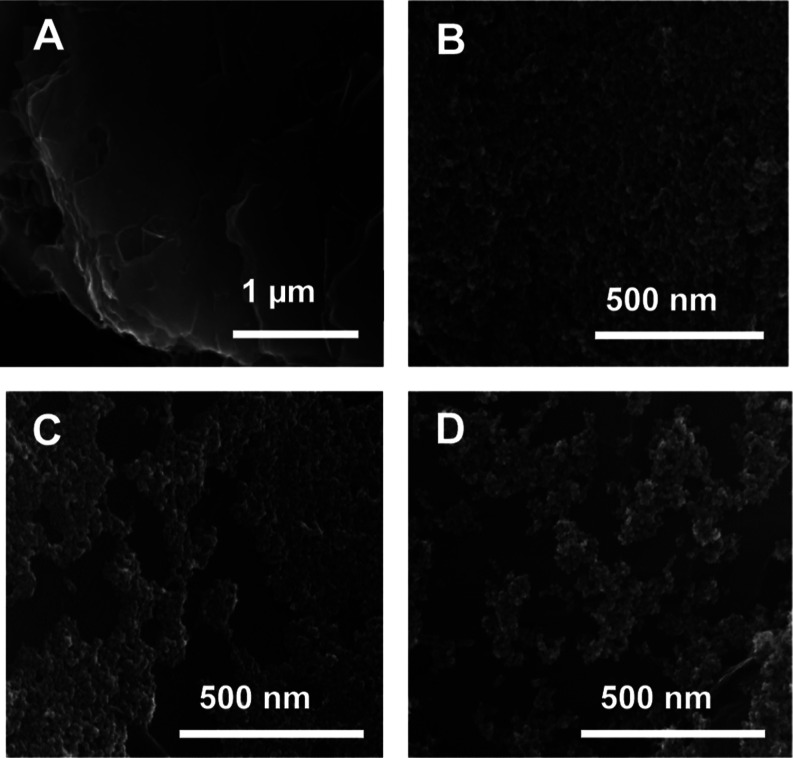
SEM images of (A) polyethylenimine (PEI) adsorbed on a high-purity graphite electrode. (B) 50, (C) 100, and (D) 200 nm MNP films adsorbed onto PEI-modified graphite electrodes.

### 3.6 Electrocatalytic Properties

Magnetite and its nanocomposites are often referred to as “nanozymes” due to their peroxidase-like enzymatic catalytic activity, which resembles that of heme proteins, catalase, and peroxidases.^10,11,19^ To investigate the electrocatalytic properties and substrate diffusion behavior of MNPs, we studied one noncatalytic substrate [a 5 mM mixture of Fe­(CN)_6_^4–^/^3–^] and two catalytic substrates (O_2_ and H_2_O_2_) dissolved in phosphate-buffered electrolytes. These studies were conducted using the RDV technique at an electrode rotation rate of 500 rpm (Figure 7A–C).

**Figure 7:**
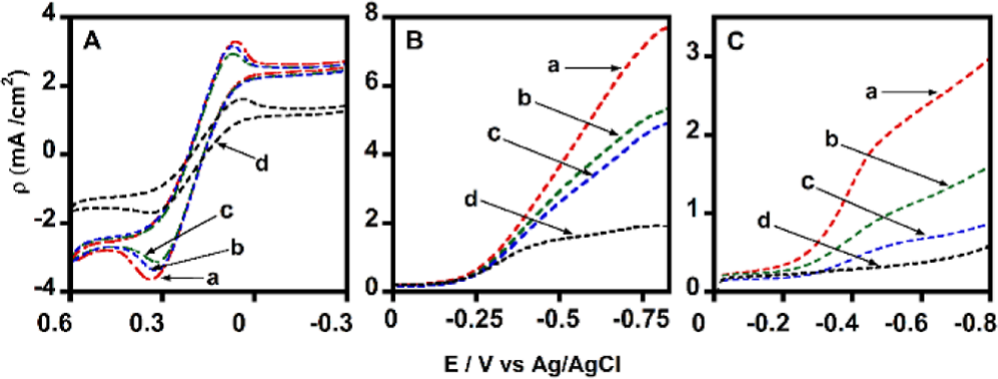
Limiting current densities from the rotating disc voltammograms of (a) 50 nm MNP, (b) 100 nm MNP, (c) 200 nm MNP, and (d) only polyethylenimine (PEI) adsorbed graphite electrodes. (A) Noncatalytic Fe­(CN)_6_^3–/4–^ mixture (5 mM each), (B) electrocatalytic saturated O_2_ reduction, and (C) H_2_O_2_ reduction (0.5 mM) in phosphate buffer (pH 7.0) at 500 rpm electrode rotation rate to facilitate the mass transport of substrates, scan rate 0.3 V s^–1^, 25 °C.

The Fe­(CN)_6_^4–/3–^ mixture showed no significant change in currents, regardless of the size of the MNPs [Figure 7A­(a–c)]. In contrast, the electrocatalytic currents from the reduction of O_2_ [Figure 7B­(a–c)] and H_2_O_2_ [Figure 7C­(a–c)] were distinct among the particles, with smaller MNPs exhibiting higher reduction currents than larger ones. The noncatalytic nature of the Fe­(CN)_6_^4–/3–^ electrochemistry at positive potentials, far from the negative iron center potential, likely explains the similar currents observed across electrodes with varying particle sizes. In this case, the magnetic particles do not appear to influence the extent of the currents generated by the Fe­(CN)_6_^4–/3–^ mixture.

The observed differences in electrocatalytic activity for O_2_ and H_2_O_2_ are directly correlated with the estimated electroactive amounts of MNPs of each size, as determined from the anaerobic cyclic voltammograms acquired in saturated argon (Table 1). The catalase-like activity of magnetite nanoparticles in a neutral medium can be explained by the mechanism described in previous work.^34^

The electrocatalytic CV cycle was designed to expose the MNP-adsorbed electrodes to the electrolyte solution for about 20 s to mitigate or avoid significant desorption-driven MNP loss from the electrode surface. This was verified through CV scans of the prepared MNP-adsorbed PEI-modified electrodes in an argon atmosphere at 4 min intervals over 20 min. The results showed an approximate 10% loss in initial currents for 50 and 100 nm MNP-adsorbed electrodes, and a more substantial loss of about 35% for 200 nm MNP-adsorbed electrodes (Figure S1). These findings suggest that for electrostatically adsorbed MNP electrodes, shorter electrolyte exposure durations of a few minutes are recommended to prevent significant nanoparticle loss due to leaching. Additionally, the increase in peak currents with increasing scan rates and the particle size-dependent electrocatalytic currents, which are directly proportional to their electroactive amounts, confirm that rapid electrocatalytic scans are effective for designing and utilizing MNP-adsorbed PEI-modified graphite electrodes.

## 4 Conclusions

This study characterized the surface arrangements and electrochemical properties of magnetite nanoparticles (MNPs) of varying hydrodynamic sizes. A comparison of electrochemical properties with microscopic images revealed that as the hydrodynamic size of MNPs increases, smaller MNPs with a greater number in contact with the electrode surface transform into larger, isolated clusters, leaving more vacant electrode surfaces and resulting in lower electroactive amounts. This interconnected network of smaller nanoparticles could enhance charge transfer with the electrode and the overall electrochemical performance. Additionally, the study highlighted the electrochemical kinetics of MNPs, characterized by slower, scan-rate-dependent forward reduction kinetics, resulting in greater peak shifts, and faster reverse oxidation kinetics, which yield smaller or negligible peak shifts with increasing scan rate. In summary, the interplay between particle size and uniform surface coverage is likely the cause of the superior electrochemical activity of smaller Fe_3_O_4_ nanoparticles. These findings highlight the crucial role of the size and spatial arrangement of magnetic materials on electrodes in designing efficient nanostructured electrode materials.

## Supplementary Material



## Notes

Table S1: hydrodynamic size and zeta potential (ζ) of poly­(acrylic acid) matrix-covered MNPs. Figure S1: stability measurements of electrostatically adsorbed films of MNPs on PEI-adsorbed HPG electrodes (PDF)
