# Supplementary material for: Size-Dependent Electrochemical and Morphological Properties of Magnetite Nanoparticles Adsorbed on Electrodes
Source: ACS Meas Sci Au. 2025 Apr 7;5(3):325–31. doi: 10.1021/acsmeasuresciau.5c00014 (PMC12183578; doi:10.1021/acsmeasuresciau.5c00014)
Supplement: Supplementary file 1 [file tg5c00014_si_001.pdf]

Supporting Information

Size-Dependent Electrochemical and Morphological Properties of Magnetite Nanoparticles Adsorbed on Electrodes

Gayan Premaratne,[a,‡] *Silan Bhandari,*[a,‡] *Charuksha Walgama,*[b,*] *Bhaskara V Chikkaveeraiah,*[c] *Albert Jin,*[c] *Sadagopan Krishnan,*[a,*]

[a] Department of Chemistry, Oklahoma State University, Stillwater, OK-74078, USA.

[b]Department of Physical & Applied Sciences, University of Houston-Clear Lake, 2700 Bay Area Boulevard, Houston, TX-77058, USA

[c] Laboratory of Cellular Imaging and Macromolecular Biophysics, National Institutes of Health, Bethesda, MD-20892, USA.

Table S1. Hydrodynamic size and Zeta potential (ζ) of polyacrylic acid-covered magnetite core nanoparticles used in this study.

**Supplier's (ChemiCell Inc.) specification/nm**	**Measured hydrodynamic diameter/ nm**	**ζ-Potential/ mV**
50	59 ± 4	-42 ± 0.2
100	108 ± 6	-39 ± 0.1
200	175 ± 10	-31 ± 0.1

As a quality control comparison with another unrelated amine-functionalized magnetic nanoparticles (amino silane matrix with a magnetite core) purchased from the same ChemiCell Inc. were also measured and agreed with the supplier specification of 100 nm hydrodynamic diameter (109 ± 1 nm with a positive ζ-potential of 24.6 ± 1.0 mV, due to the surface amine positive

groups in phosphate buffer, pH 7.0, N=3 replicates) as against the negative ζ-potential of polyacrylic acid-covered MNPs.

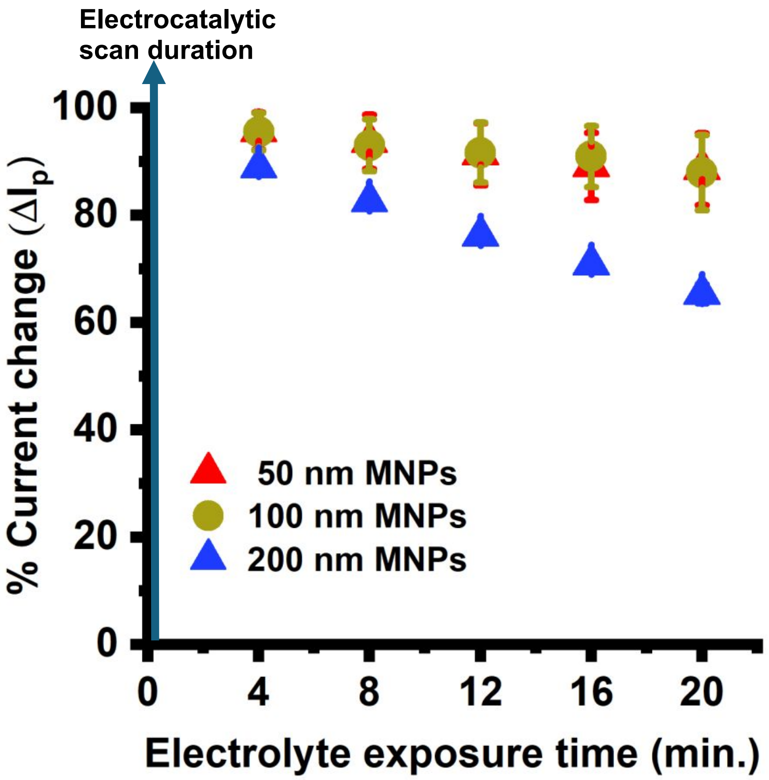

Figure S1. The decrease in the reduction peak current was used as a probe to estimate the stability of the electrostatically adsorbed films of MNPs on PEI polycation-modified HPG electrodes with exposure time in the electrolyte solution, phosphate buffered solution (pH 7.0, 0.1 M KCl), saturated argon, 25 ^{o}C, Scan rate 0.2 Vs^{-1}.
